# *Leptospermum flavescens* Constituent-LF1 Causes Cell Death through the Induction of Cell Cycle Arrest and Apoptosis in Human Lung Carcinoma Cells

**DOI:** 10.1371/journal.pone.0135995

**Published:** 2015-08-19

**Authors:** Suerialoasan Navanesan, Norhanom Abdul Wahab, Sugumaran Manickam, Kae Shin Sim

**Affiliations:** 1 Institute of Biological Sciences, Faculty of Science, University of Malaya, Kuala Lumpur, Malaysia; 2 Biology Division, Centre for Foundation Studies in Science, University of Malaya, Kuala Lumpur, Malaysia; 3 Rimba Ilmu Botanic Garden, Institute of Biological Sciences, Faculty of Science, University of Malaya, Kuala Lumpur, Malaysia; Taipei Medicine University, TAIWAN

## Abstract

*Leptospermum flavescens* Sm. (Myrtaceae), locally known as ‘Senna makki’ is a smallish tree that is widespread and recorded to naturally occur in the montane regions above 900 m a.s.l from Burma to Australia. Although the species is recorded to be used traditionally to treat various ailments, there is limited data on biological and chemical investigations of *L*. *flavescens*. The aim of the present study was to investigate and understand the ability of *L*. *flavescens* in inducing cell death in lung cancer cells. The cytotoxic potentials of the extraction yields (methanol, hexane, ethyl acetate and water extracts as wells as a semi pure fraction, LF1) were evaluated against two human non-small cell lung carcinoma cell lines (A549 and NCI-H1299) using the MTT assay. LF1 showed the greatest cytotoxic effect against both cell lines with IC_50_ values of 7.12 ± 0.07 and 9.62 ± 0.50 μg/ml respectively. LF1 treated cells showed a sub-G_1_ region in the cell cycle analysis and also caused the presence of apoptotic morphologies in cells stained with acridine orange and ethidium bromide. Treatment with LF1 manifested an apoptotic population in cells that were evaluated using the Annexin V/ propidium iodide assay. Increasing dosage of LF1 caused a rise in the presence of activated caspase-3 enzymes in treated cells. Blockage of cell cycle progression was also observed in LF1-treated cells. These findings suggest that LF1 induces apoptosis and cell cycle arrest in treated lung cancer cells. Further studies are being conducted to isolate and identify the active compound as well to better understand the mechanism involved in inducing cell death.

## Introduction

Cancer has plagued mankind since the beginning of recorded history. It is a persistent ailment where more than a million people are diagnosed with this disease each year and has a considerably social impact and significant economic burden on our healthcare system. One form of this disease that is severely overlooked is lung cancer which accounts for 19.8% of all medically certified deaths due to cancer in Malaysia [1]. On a global perspective, new cancer diagnosis largely consists of lung cancer cases, whereby 1,350,000 or 12.4% of new cases are contributed from lung cancer [2].

Mutations in the respiratory epithelium gives rise to lung cancer which can be categorized into two broad groups, small cell lung cancer (SCLC) and non–small cell lung cancer (NSCLC). 15% of all lung cancer cases are SCLC which is a highly malignant form of tumour that originates from cells that exhibit neuroendocrine characteristics. The remaining 85% of cases fall into the NSCLC category which is broken down into 3 more groups based on their pathologic subtypes, adenocarcinoma, large cell carcinoma and squamous cell carcinoma [3]. Worldwide, NSCLC patients have a low survival rate; less than 20% of patients survived beyond 5 years after being diagnosed with the disease [4–6].

According to the World Health Organization (WHO), an increasing trend is expected in the number of new lung cancer cases in the years to come, especially in Asia. In fact, since 1985, the number of lung cancer cases has risen by 51%, mostly in females whereby a jump of 76% of new cases have occurred. Both of these incidences are based on a global increase in the usage of tobacco, which is found to be the principal risk factor for lung cancer and causes a large portion of all pulmonary carcinomas [7]. Non-smokers are also affected by this ever prominent disease, mainly through one or a combination of reasons including genetic factors [8], asbestos, air pollution [9], second-hand smoke and also radon exposure [10], which is the second major cause of lung cancer. The usage of standard chemotherapy may help to a certain extent but more often the survival benefits are at the expense of severe toxicity. Based on this reasoning, an ethical imperative arises to seek non-toxic alternative to current chemotherapy drugs that is able to effectively combat and manage this disease and at the same time exclude severe toxicity and unwanted complications that are synonymous with current treatment.

For this purpose, we turn our attention towards natural products, which is a huge repository of stereochemically complex molecules that appear to have a high specificity towards certain biological target. More than half of approved drugs available today have either been directly sourced from natural origins or are analogues to naturally occurring compounds [11].

In the current study, *Leptospermum flavescens* was chosen as the candidate for a natural product based treatment against NSCLC. *L*. *flavescens* is a medium sized shrub in the Myrtaceae family with many twiggy angular branches and a reddish-brown bark. The flowers are solitary, numerous and white with a yellow tinge. *L*. *flavescens* has been used as a natural remedy by locals, either eaten raw or taken as a concoction brewed from fresh plants. In South East Asia, *L*. *flavescens* is used to treat constipation, lethargy, hypertension, diabetes and kidney pains [12]. The leaves are used to stimulate appetite and a decoction of the leaves is drunk for relief of stomach discomfort and dysmenorrhoea [13]. The volatile oil extracted from the leaves is used as a remedy to relieve bronchitis when inhaled and also used as an embrocation for rheumatism. Although *L*. *flavescens* is reported to be used in traditional medicine preparations, there is limited recorded data on the biological and chemical investigations of the species. At the time of writing, there has been no specific evaluation of the anti-tumour properties against lung cancer cells. Demuner *et al*. [14] studied the chemical composition as well as the antimicrobial activities of the essential oil from this plant. The study showed that nerolidol was a major component of the extracted volatile oils with a higher antimicrobial activity against Gram-positive bacteria compared to Gram-negative bacteria.

The aim of this study was to evaluate the cytotoxic effect of *L*. *flavescens* against selected NSCLC. The ability of the active fraction to induce apoptosis and disrupt the cell cycle progression was also assessed in this study. The study managed to fulfill these objectives by showing that *L*. *flavescens* possess the capacities of eliminating the cancer cell in a controlled manner through the apoptotic pathway.

## Materials and Methods

### Plant sample collection and identification

Fresh plant sample of *L*. *flavescens* was collected from Genting Highlands, Pahang, Malaysia on April 2012. No specific permissions were required for collecting samples in this location. The area of collection is accessible to general public and not designated as a specific area where permission is needed for collection such as a nature park or a wildlife sanctuary. The species is also not an endangered plant or a protected species as it is generally found established on elevations of around 1200 m or higher throughout Malaysia. The plant was identified by Dr Sugumaran Manickam of Institute of Biological Sciences, Faculty of Science, University of Malaya, Malaysia with a voucher specimen number: KLU 47798. The voucher specimen was deposited at the herbarium of the Institute of Biological Sciences, Faculty of Science, University of Malaya, Kuala Lumpur, Malaysia.

### Extraction of extracts and LF1

The collected plant samples were washed, dried and ground into a fine powder. The dried and ground plant materials (1000.00 g) were extracted with methanol for three days at room temperature to obtain crude methanol extract (163.00 g, 16.3%). Upon reserving a small portion (10.00 g) for testing, the remainder (153.00 g) was subjected to fractionation with hexane to yield hexane-soluble extract (6.30 g, 4.12%) and hexane-insoluble residue. The residue was further partitioned with ethyl acetate and water (1:1, 100 ml: 100 ml) to obtain an ethyl acetate (30.00 g, 19.61%) and a water (29.40 g, 19.22%) extract. A semi-pure fraction (LF1; approx. 12.6 g) which was a white powder with slight greenish tinge was precipitated during methanol extraction, and was included in the current study together with the crude methanol and fractionated (hexane, ethyl acetate and water) extracts. All extracts and LF1 were weighed and stored in a fridge (4°C) before further experimentation was done. Prior to testing, all the extracts were dissolved in dimethyl sulfoxide (DMSO), with the exception of the water extract which was dissolved in distilled water to form the stock solution.

### Cell lines and culture medium

The A549 (ATCC CCL-185) and NCI-H1299 (ATCC CRL-5803) human non-small cell lung carcinoma cell lines and MRC-5 (ATCC CCL-171) normal human lung fibroblast cell line were purchased directly from American Type Culture Collection (ATCC, Manassas, VA, USA). The A549 and NCI-H1299 cells were maintained in a RPMI 1640 medium while MRC-5 cells in an EMEM medium. All media were supplemented with 10% foetal bovine serum, 1% penicillin/streptomycin (100×) and 0.5% amphotericin B while addition of 1% sodium pyruvate and 20% foetal bovine serum was supplemented for the EMEM medium. The cells were cultured at 37°C in a Shellab humidified CO_2_ incubator (Sheldon Manufacturing, Inc., Cornelius, OR, USA).

### MTT cytotoxicity assay

The cytotoxicity of extracts and LF1 were assessed using MTT (3-(4, 5-dimethylthiazolyl-2)-2, 5-diphenyltetrazolium bromide) assay as previously described by Mosmann [15] with modifications. The cells were seeded at a concentration of 7,000 cells per well in a 96-well plate a day prior to treatment with the extracts and LF1 that were diluted to contain 0.5% DMSO. The treated cells were left to incubate for 72 hours in the CO_2_ incubator. Addition of the MTT solution (working concentration of 5 mg/ml) was followed by a further incubation period of 3 hours before dissolving the purple formazan crystals with DMSO. The absorbance values was measured using a Multiskan GO micro plate spectrophotometer (Thermo Fisher Scientific Inc., Waltham, MA, USA) at 570 nm with 650 nm as a reference wavelength. Cisplatin was used as positive reference standard. All assays were performed in triplicates.

### Acridine Orange / Ethidium Bromide (AO/EB) double staining assay

The cytotoxic extracts were assessed using the AO/EB staining technique as previously described by Ribble *et al*. [16]. The cells were seeded at a concentration of 100,000 cells per well in a six-well plate and incubated for 24 hours at 37°C before treatment with the active fraction LF1 at varying concentrations (5, 15 and 25 μg/ml). Following an incubation period of 24 hours, the cells were detached and pelleted. The supernatant was removed and the cells were subsequently stained with the prepared dye mixture (25 μl cold PBS and 2 μl EB/AO dye mixed in a 1:1 ratio). The stained cell suspension was transferred onto a clean glass slide and covered with a coverslip. The morphological changes relative to the untreated control was observed using the narrow blue excitation filter on an Olympus IX73 fluorescent microscope (Olympus Corporation, Shinjuku, Tokyo, JPN). The images were photographed at ×400 magnification.

### Annexin V/ Propidium Iodide (PI) assay

The Annexin V-FITC/ PI assay was conducted to further assess the effects of the cytotoxic fraction LF1 in inducing cell death through apoptosis. The cells were plated at a concentration of 300,000 cells per dish on a 60 mm culture dish and incubated for 24 hours at 37°C before being treated with a few different concentrations of LF1. In order to determine the induction of apoptosis, cells were harvested at 24, 48 and 72 hours post treatment. The cells were detached, washed with phosphate buffered saline (PBS) and stained with Annexin V-FITC conjugates and Propidium Iodide (PI) (BD Biosciences, San Jose, CA, USA). The cells were incubated for 15 minutes before they were analysed using a BD FACS Canto II flow cytometer (BD Biosciences, San Jose, CA, USA). A minimum of 10,000 events were collected and analysed.

### Active Caspase-3 detection assay

LF1 induced apoptosis was ascertained by detecting the presence of activated caspase-3 in treated cells. The FITC Active Caspase-3 Apoptosis Kit (BD Biosciences, San Jose, CA, USA) was used to assess the presence of active caspase-3. The antibody used in this assays specifically recognize the active form of caspase-3 in human cells. The cells were plated at a concentration of 300,000 cells per dish on to a 60 mm culture dish and left for 24 hours at 37°C before addition of treatment at predetermined concentrations. The cells were then left to incubate for another 24 hours before being harvested and stained according to the manufacturer’s protocol. The cells were then analysed using the BD FACS Canto II flow cytometer (BD Biosciences, San Jose, CA, USA) with a minimum of 10,000 events being acquired.

### Cell cycle analysis

The presence of cell cycle disruption caused by the treatment of the cytotoxic fraction LF1 was evaluated using BD Cycletest Plus DNA Reagent Kit (BD Biosciences, San Jose, CA, USA). The cells were plated at a concentration of 300,000 cells per dish on to a 60 mm culture dish and left for 24 hours at 37°C before addition of treatment at predetermined concentrations. The cells were then left to incubate for another 24 hours before being harvested. The kit was then used for the isolation and staining of nuclei in treated cells. The PI stained nuclei was then analysed using the BD FACS Canto II flow cytometer (BD Biosciences, San Jose, CA, USA). The DNA samples were acquired at a low flow rate to ensure the best resolution for the collected data. A minimum of 10,000 events were collected and analysed. The sub-G_1_ was gated and quantified using the FACS Diva software (Beckton-Dickinson, USA) while the cell cycle data was analysed using the ModFit software (Verity Software House, Topsham, ME, USA).

### Statistical analysis

The IC_50_ values of cytotoxic activity were obtained by non-linear regression using GraphPad Prism 5 software (GraphPad Software Inc., USA). The software was also used to perform a Dunnett's Multiple Comparison Test on the results from the Annexin V-FITC/PI assay, cell cycle, sub-G_1_ analysis and active caspase-3 detection assay to determine if the variation from the untreated control is statistically significant.

## Results

### Cytotoxic activity of *L*. *flavescens* extracts and LF1

The cytotoxicity of the crude methanol extract, its fractions (hexane, ethyl acetate and water) and the LF1 fraction was assessed using the MTT cytotoxic assay described in the methodology section. The samples were tested on three different lung cells line (A549, NCI- H1299 and MRC-5). The results obtained were presented in Table 1. An extract is considered significantly cytotoxic should it produce an IC_50_ value ≤ 20 μg/ml. Based on this criteria, none of the crude and fractionated extracts managed to exert a significant cytotoxic affect against all three cell lines. The semi-pure fraction, LF1, managed to produce and IC_50_ value of 7.12 ± 0.07 and 9.62 ± 0.50 μg/ml against the population of A549 and NCI-H1299 respectively. The cells were also tested using an FDA approved drug for the treatment of lung cancer, cisplatin. An IC_50_ value of 8.70 ± 1.30 μg/ml for A549 and 21.41 ± 3.99 μg/ml against NCI-H1299 were obtained. Cisplatin seemed to be more toxic towards the normal fibroblast cell, MRC-5, compared to LF1 (1.27 ± 0.06 μg/ml against 6.66 ± 0.17 μg/ml).

**Table 1:** Cytotoxic activity (IC_50_ values) of extracts.

Extracts/Fraction	Cytotoxicity (IC_50_) in μg/ml		
	A549	NCI-H1299	MRC-5
Methanol	96.38 ± 4.03	> 100	> 100
Hexane	40.91 ± 0.97	90.44 ± 1.55	69.11 ± 5.15
Ethyl Acetate	55.77 ± 2.59	77.10 ± 2.81	62.20 ± 1.45
Water	> 100	> 100	> 100
LF1	7.12 ± 0.07	9.62 ± 0.50	6.66 ± 0.17
Cisplatin *	8.70 ± 1.30	21.41 ± 3.99	1.27 ± 0.06

*Positive reference standard. Values are expressed as mean ± standard deviation (n = 3).

### Morphological detection of cell death caused by LF1 using AO/EB double staining

**Fig 1:**
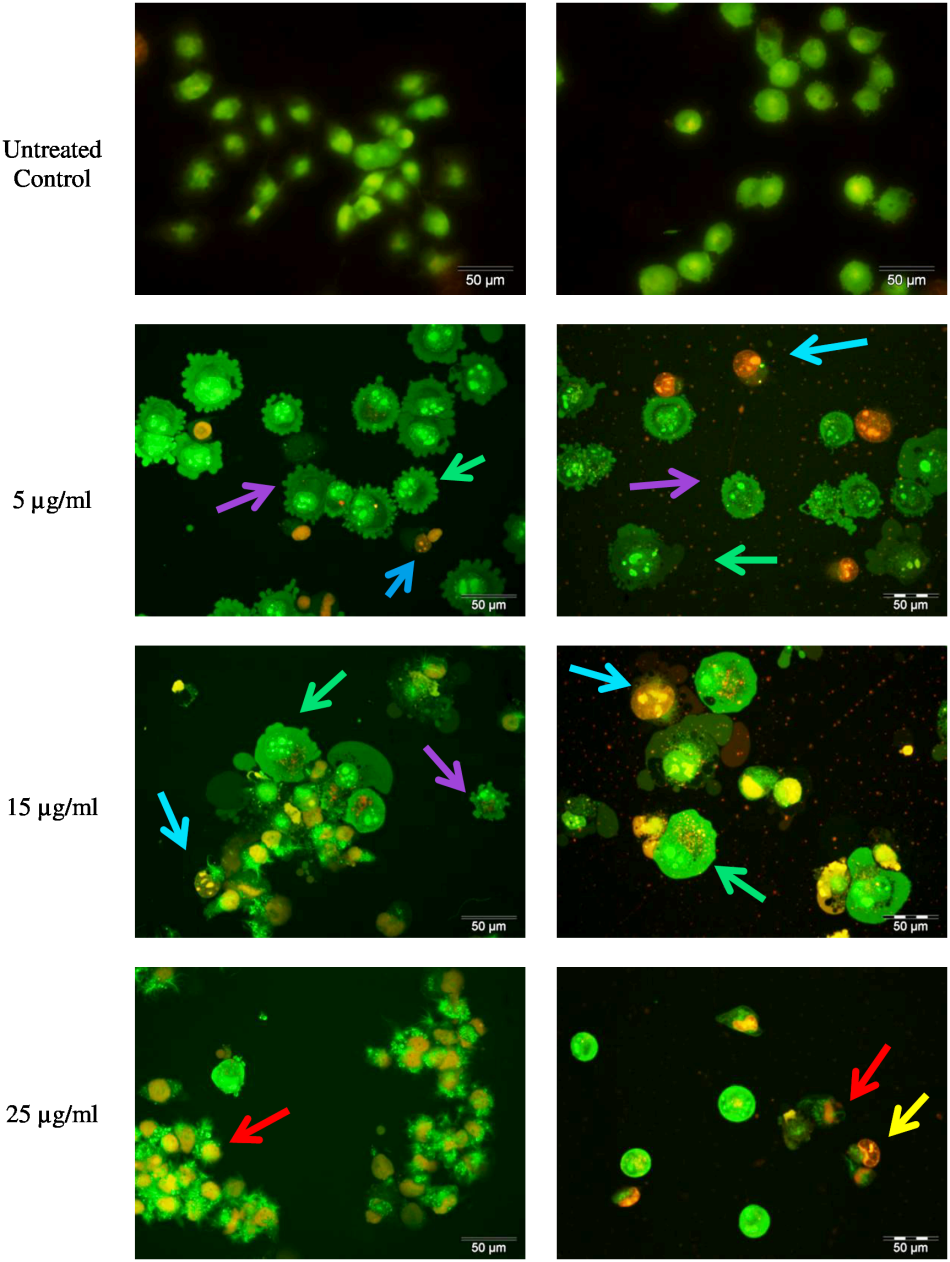
Morphological observation of LF1 treated A549 and NCI-H1299 using AO/EB staining at × 400 magnifications. A549 and NCI-H1299 were treated without (untreated control) and with LF1 at different concentrations. Green arrows indicate early apoptotic cells (chromatin condensation stained green); blue arrows are late apoptotic cells (chromatin condensation stained orange); purple arrows shows membrane blebbing; yellow arrows are for cells which appear shrunken and red arrows indicate loss of membrane shape. The higher the concentration of LF1 used, the more aggressive pathway taken in the induction of death in the cancer cells.

LF1 treated cells showed morphological features consistent with apoptosis. In comparison with the untreated cells which produced a nuclei uniformly stained green, LF1 treated A549 and NCI-H1299 cells showed bright patches of bright green fluorescent stains within the cells (Fig 1), indicative of chromatin condensation. Moreover, at concentration of 5 μg/ml of LF1, both cell lines produced protrusion out of the plasma membrane which could be characterized as membrane blebbing, one of the symptoms commonly associated with early apoptosis. With the increase in dosage of LF1 used, an elevation in the number of cells stained orange is also observed. Treatment with the highest concentration (25 μg/ml) of LF1 appears to cause the cells to be uniformly stained orange. The cells also tend to appear to have a decreased definition of the nuclear outline, possibly due to the rupturing of nuclear and plasma membrane.

### Sub-G_1_ population analysis of LF1 treated cells

**Fig 2:**
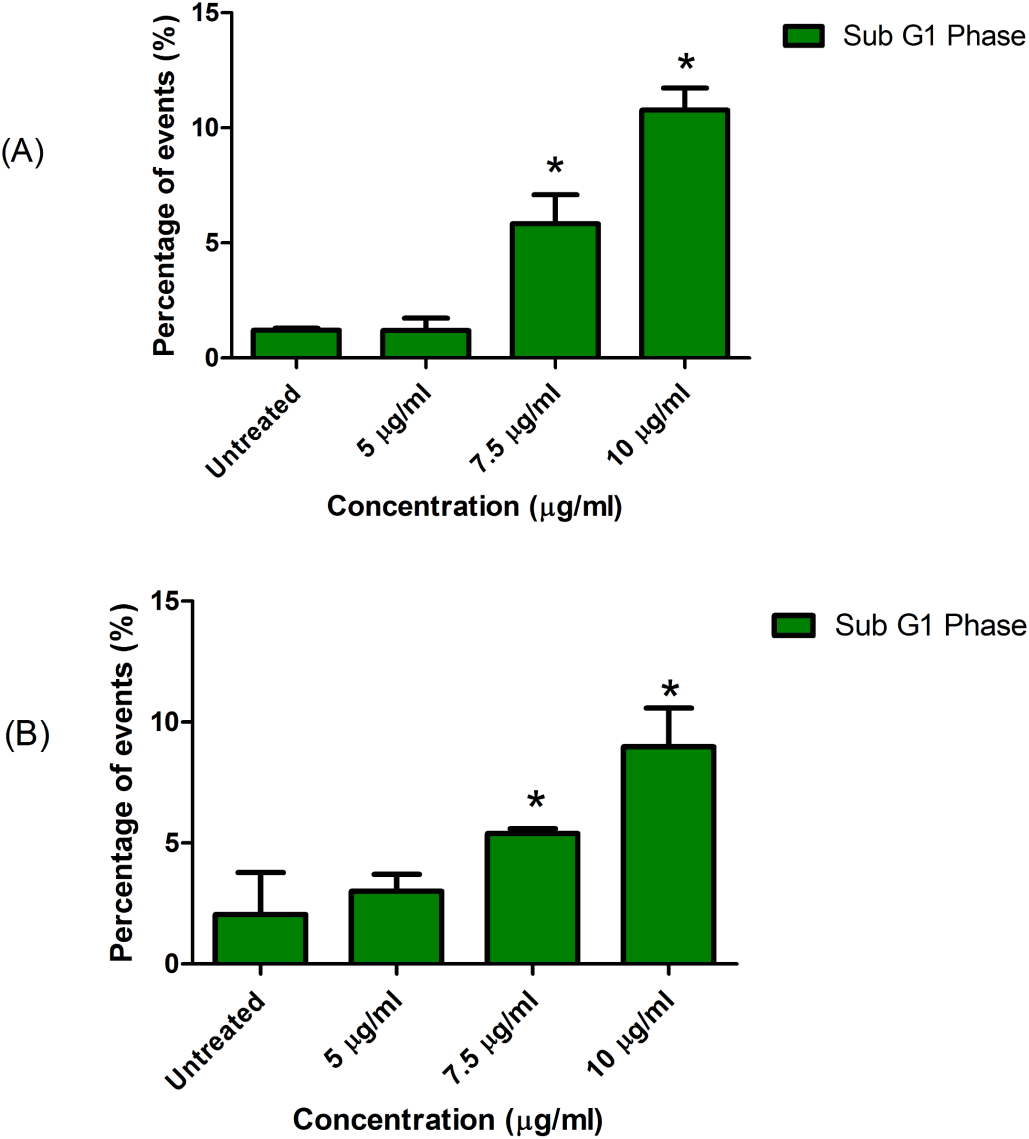
Sub G_1_ population of LF1 treated cells. A549 (A) and NCI-H1299 (B) cells were treated with 5, 7.5 and 10 μg/ml of LF1. The resultant sub-G_1_ population show an increase with the increase in concentration of LF1 used.

Quantification of the Sub-G_1_ population was done concurrently with the cell cycle analysis which utilized the PI dye. The results were expressed as percentage relative to the total acquired gated-population using the flow cytometer. As the concentration of LF1 is increased, the quantity of cells which produces a Sub-G_1_ peak in DNA frequency histogram also gradually increases (Fig 2). The increment is significantly (p<0.05) prominent upon treatment with 7.5 and 10 μg/ml of LF1. This situation is observed in both A549 and NCI-H1299 cell lines. The percentage of sub-G_1_ population increases from 1.20 ± 0.10% in untreated cells to 10.77 ± 0.97% in A549 cells treated with 10 μg/ml of LF1, while NCI-H1299 cells exposed to similar conditions increased from 2.05 ± 1.73% to 8.98 ± 1.59%.

### Assessing LF1-induced apoptosis using Annexin V-FITC /Propdium Iodide

**Fig 3:**
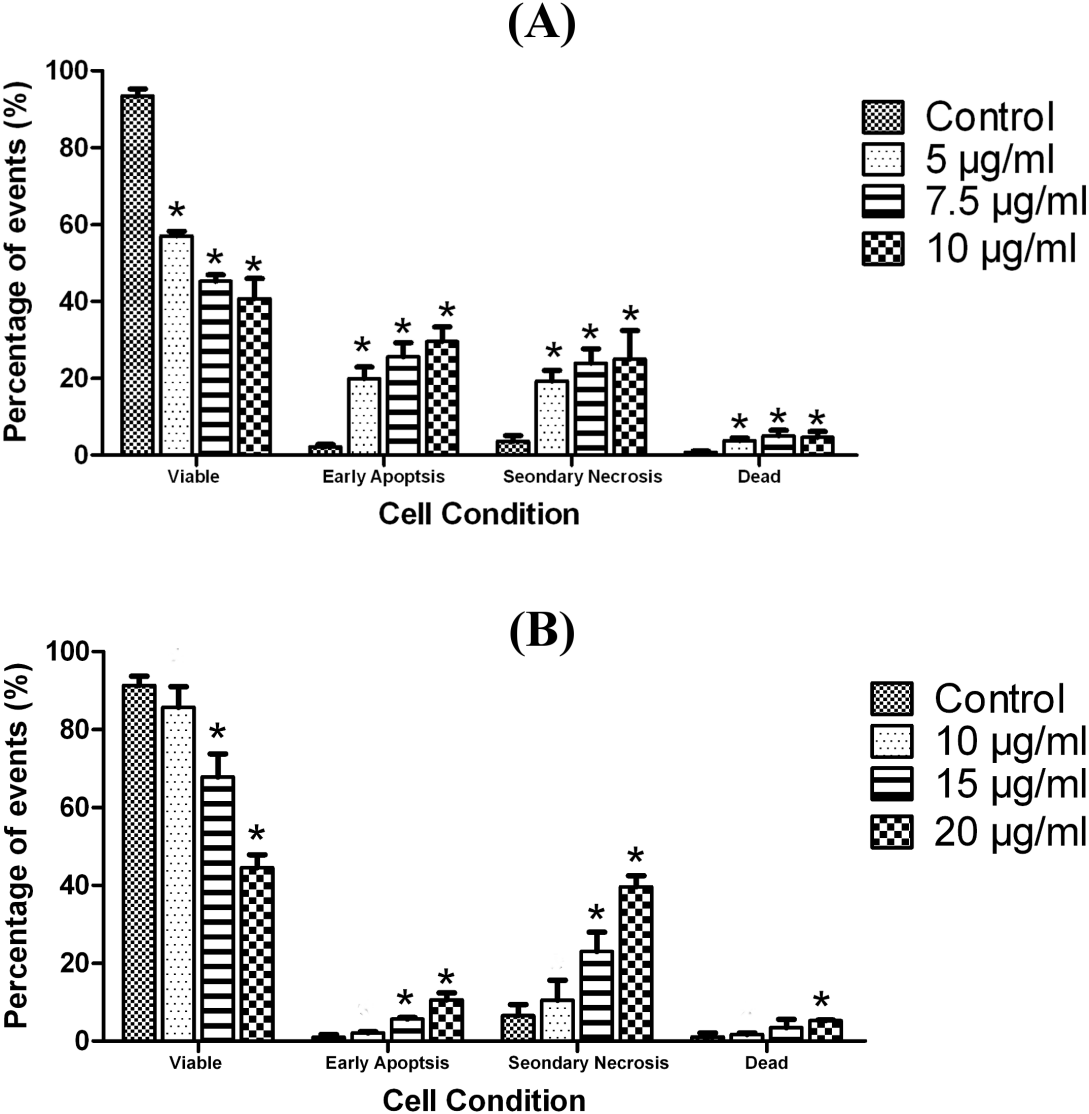
Effects of exposure to LF 1 for 24 hours on the externalization of PS in A549 and NCI-H1299 (Dose—dependent). Summary of the results for 24 hours treatment with LF1 was presented in a bar chart (A) for A549 and bar chart (B) for NCI-H1299. A significant increase in the early apoptotic and secondary necrotic cell population is observed with the increasing concentration of LF1 in both cell lines.

**Fig 4:**
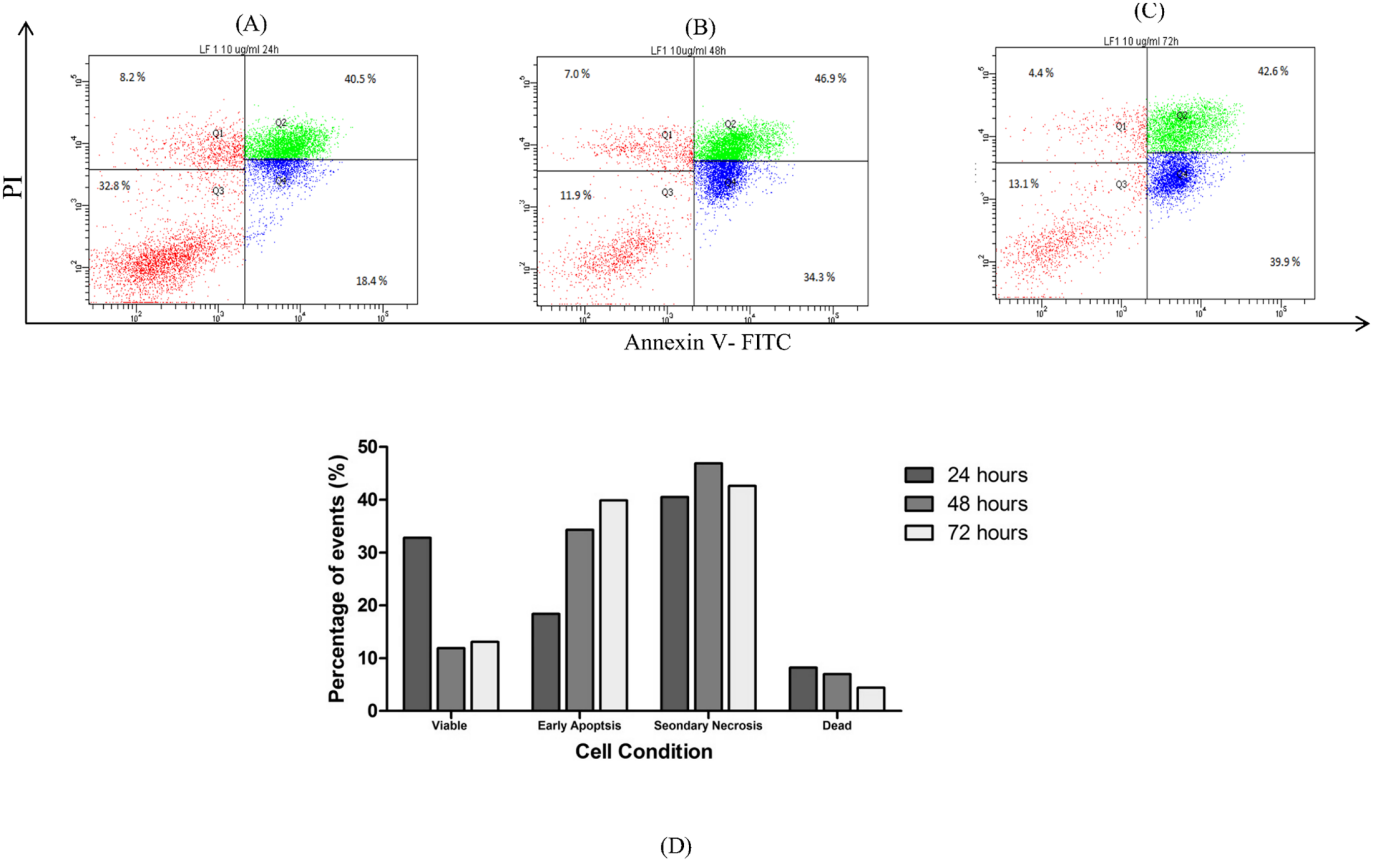
Effects of exposure to LF1 on the externalization of PS in A549 at different time points (Time—dependent). Cells were treated with 10μg/ml of LF1 for 24 hours (A), 48 hours (B) and 72 hours (C). Summary of the results were presented in a bar chart (D). An increase in early apoptotic cells over time that was then followed by an increase in double positively stained cells indicate an apoptotic pathway was taken by LF1 treated cells.

**Fig 5:**
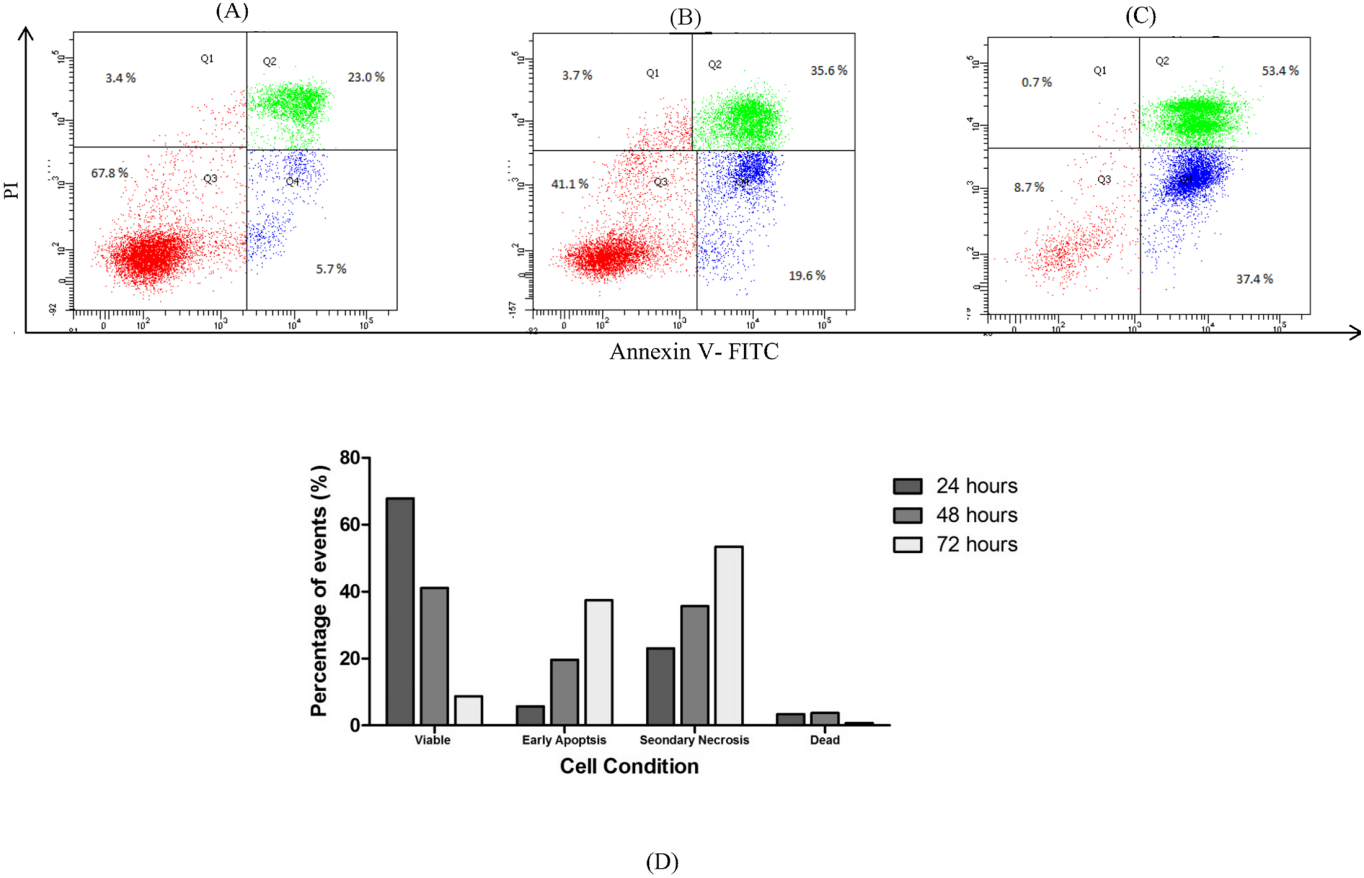
Effects of exposure to LF1 on the externalization of PS in NCI-H1299 at different time points (Time—dependent). Cells were treated with 15 μg/ml of LF1 for 24 hours (A), 48 hours (B) and 72 hours (C). Summary of the results were presented in a bar chart (D). An increase in early apoptotic cells over time that was then followed by an increase in double positively stained cells indicate an apoptotic pathway was taken by LF1 treated cells.

Incidence of apoptosis in cells exposed to LF1 was quantified using the Annexin V-FITC/Propidium Iodide assay. Flow cytometric analysis of A549 and NCI-H1299 cells stained with Annexin V-FITC conjugates and PI expressed a significant (p < 0.05) increase in the early apoptotic and secondary necrotic cell population with the increasing concentration of LF1 used (Fig 3). With reference to the untreated control, the number of viable cells suffered a steep drop in population for both cell lines used; from 93.4 ± 1.9% to 40.7 ± 5.3% for A549 and 91.4 ± 2.3% to 44.6 ± 3.3% for NCI-H1299 (Fig 3). These observations were done with reference to the untreated controls and the differences with the control were statistically significant (p < 0.05) in most cases. In order to distinguish between secondary necrotic cells and necrotic cells, a time course study was performed. Both cells lines showed an increase in early apoptotic cells over time that was then followed by an increase in double positively stained cells (Figs 4 and 5), indicating that the treated cells have undergone apoptosis.

### Detecting the presence of active (cleaved) caspase-3

**Fig 6:**
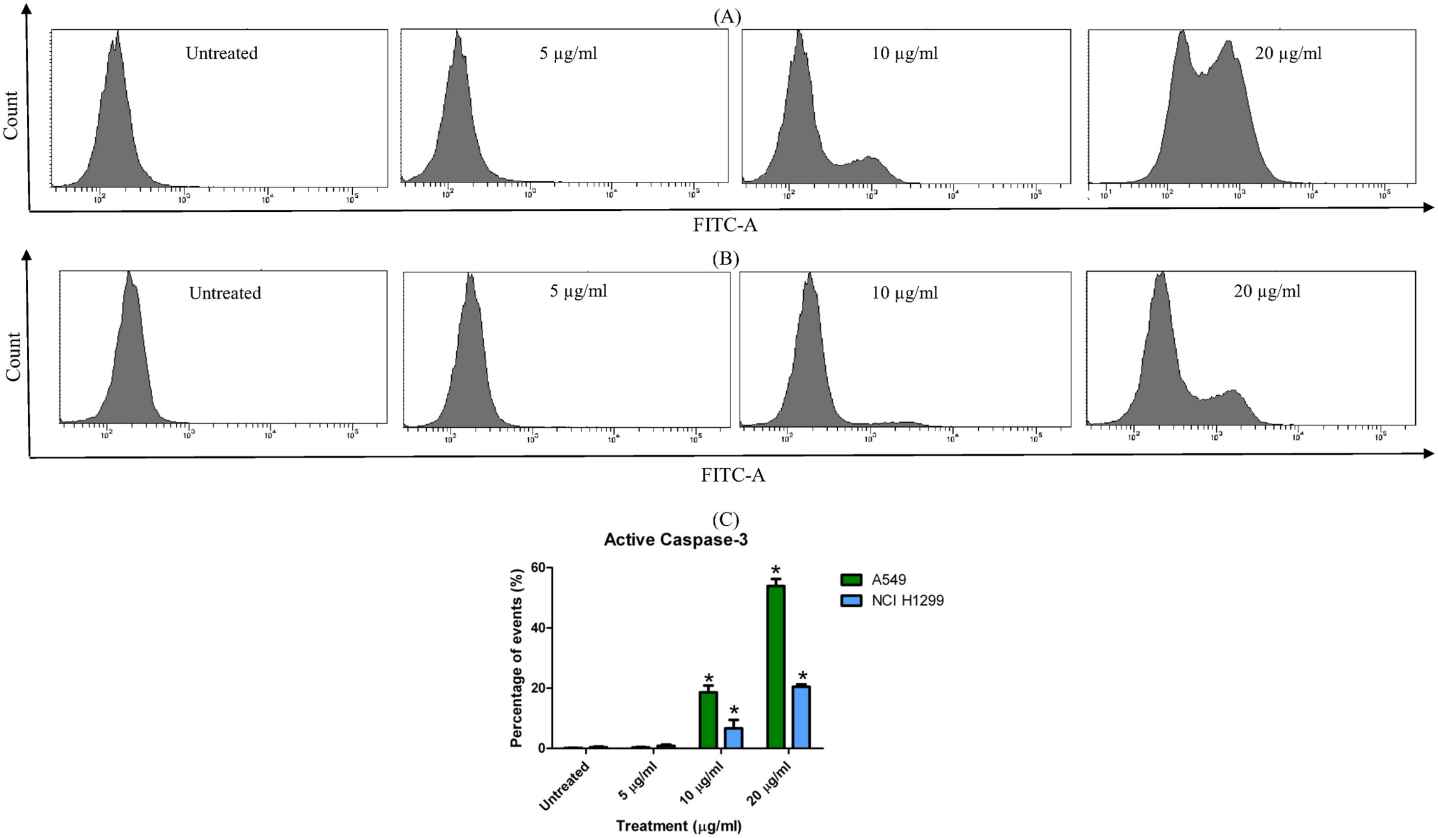
Presence of active caspase-3 in LF1 treated A549 and NCI-H1299. (A) A549 and (B) NCI-H1299 cells were treated with 5, 10 and 20 μg/ml of LF1 for 24 hours. Summary of the results were presented in a bar chart (C). An increase in the presence of cleaved caspase-3 was detected in both cell lines in a dose-dependent manner, indicating an apoptotic pathway was taken by LF1 treated cells.

The activated caspase-3 binds covalently and irreversibly with the FITC-conjugated antibody and is expressed as percentage relative to the total acquired gated-population using the flow cytometer. As shown in Fig 6, an increasing presence of activated caspase-3 is observed with the increasing treatment of LF1 in both A549 and NCI-H1299, although a stronger effect is seen in the former (Fig 6). A shift towards the right is seen in the histogram as higher dosages of LF1 are used against both cell lines, indicating a rise in cleaved caspase-3 presence. Treatment with 5 μg/ml of LF1 did not seem to elicit significant caspase activation as compared to the untreated control on both cell lines. On the other hand, a significant increase (p < 0.05) from 0.13 ± 0.15% to 53.90 ± 2.36% of active caspase-3 signal was observed in A549 treated with 20 μg/ml of LF1 whilst NCI-H1299 cells experienced a smaller jump from 0.40 ± 0.26% to 20.47 ± 0.80% under the same conditions.

### Analysing the disruptive effect of LF1 on the cell cycle

**Fig 7:**
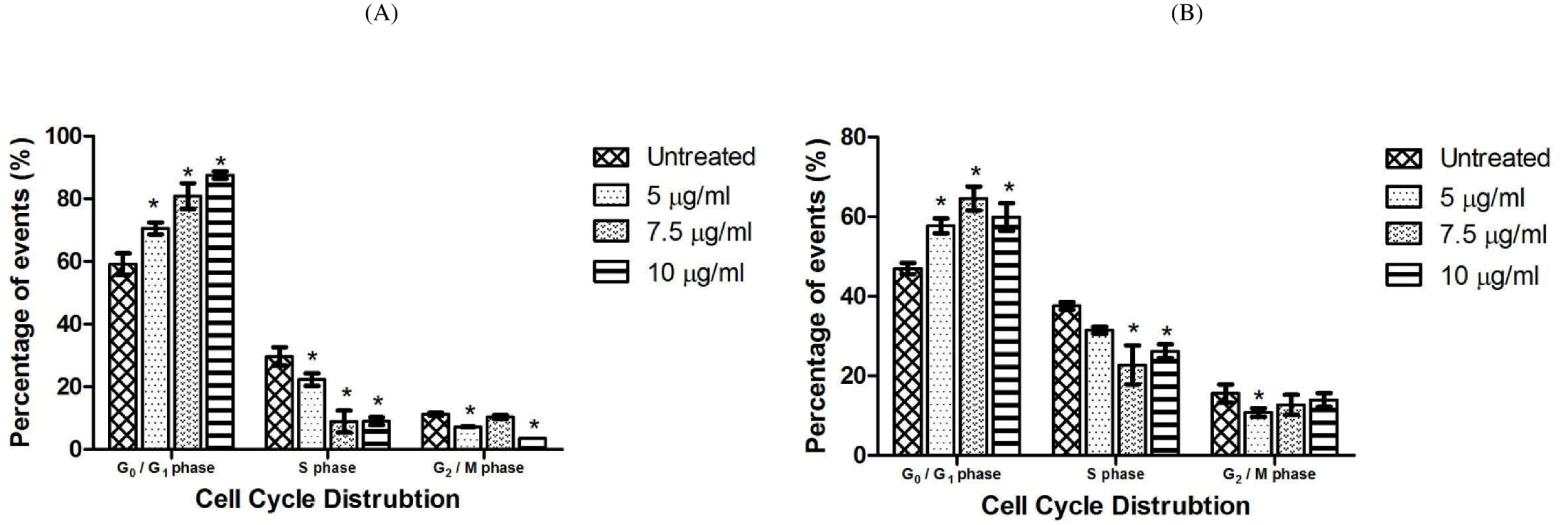
Effects of LF1 on cell cycle distribution in A549 NCI-H1299 cells. A549 (A) and NCI-H1299 (B) were incubated in absence (control) and presence of LF1 at 5, 7.5 and 10 μg/ml for 24 hours. Summary of results indicate an increase in G_0_/G_1_ population with increasing dosages of LF1 used.

Blockage in the cell cycle as a result of treatment with LF1 was assessed by staining fixed and permeabilized cell with the PI dye. The DNA content which was in the G_0_/G_1_ phase gradually increased with the increasing exposure to LF1 on both A549 and NCI-H1299 cell lines. In general, a reverse was observe in the S and G_2_/M phase population of both treated cell lines, whereby the number of events recorded depleted with the escalating dosage of LF1 used (Fig 7). These observations were done with reference to the untreated controls and the differences with the control were statistically significant (p < 0.05) in most cases.

## Discussion

The alarming rise in incidence of cancer, especially lung cancer, encourages us to look for alternative cures which are more effective in the fight against cancer but at the same time, harmless towards the patient. The purpose of this study was to explore the usage of *Leptospermum flavescens* to fulfill the above needs.

The MTT assay is a mode of convenient quantitative measurement used in evaluating a population of cell’s response to external factors which may cause an increase or decrease in cell growth [17]. In this study, the crude methanol and fractionated (hexane, ethyl acetate and water) extracts as well as the semi-pure fraction LF1 were tested against three lung cells, two cancerous and one normal cell line. The comparable characteristic of the cancerous cells used is that A549 has a functional wild type p53 expression while NCI-H1299 is p53 deficient. The p53 functions by coordinately blocking cell proliferation, stimulating DNA repair and promoting apoptotic cell death in damaged cells [18]. A normal human lung fibroblast cell line (MRC-5) was used to determine the effect on non-carcinoma cells. The criteria set forward by US National Cancer Institute (NCI) Plant Screening Program was that a compound is incubated with the cancer cells between 48 and 72 hours, it should produce an IC_50_ value less than 20 μg/ml for a crude extract and 4 μg/ml for a pure compound [19]. With reference to Table 1, the toxicity towards both the cancer cell lines tested indicates that there is a possibility that the fraction has utilized a p53 independent pathway in inducing cell death as a similar cytotoxic effect was present regardless of the presence of a functional p53 gene expression.

Although LF1 may seem to be toxic towards the normal MRC-5 cell, the positive control used (cisplatin), which is a drug approved by US Food and Drug Administration (FDA) for usage in lung cancer treatment, exhibited a much more potent effect towards the normal fibroblast cell. In fact, LF1 was five times less toxic towards the normal cells when compared to cisplatin. Based on these results, the ability of LF1 to induce cell death was further assessed.

LF1 was further examined using the AO/EB double staining method. This combination of dyes is commonly applied in the identification and differentiation of live, apoptotic and necrotic cells. Cells are differentiated through the staining pattern. Viable cells are stained uniformly green whereas early apoptotic cells have bright green patches, indicative of chromatin condensation. Late apoptotic cells have orange to red stained nuclei with condensed or fragmented chromatin and necrotic cells have a uniformly stained orange to red nuclei [20]. Morphological observations commonly associated with apoptosis such as membrane blebbing and fragmented nuclei were used to classify the cells as apoptotic or otherwise [21].

By taking into account the nuclear morphology visualized through the fluorescent AO/EB stain (chromatin condensation, nuclear collapse) and the common light microscopy appearances (membrane blebbing, cell rounding and shrinkage) of these cells, a complete profile of an apoptotic cells is present. These observations point towards the interpretation that LF1 treated cells undergo apoptosis which will lead to the eventual death of the cells.

The observations during treatment with the highest concentration vary considerably with the pattern seen in the other treatment groups. The uniformly stained orange nucleus suggests that at high concentration of LF1, the cells undergo necrosis instead of apoptosis. According to Sancho-Martinez *et al*. [22], the concentration of many cytotoxic molecules and stimuli, including different anticancer drugs, was the determinant in the manner of cell death, being either apoptosis or necrosis. The high concentration of the drug which caused necrosis in the treated cells could have been caused by general toxicity of foreign compound towards the cells. In short, the higher the concentration of LF1 used, the more aggressive pathway taken in the induction of death in the cancer cells.

To further prove the hypothesis that LF1 is able to induce cell death in cancer cells through apoptosis, presence of a sub-G_1_ population in the DNA content frequency histogram was investigated. Cells that are or have undergone apoptosis should contain fractional DNA content which translates into the presence of a hypo-diploid DNA population that lies before the diploid (2N) region in the DNA content frequency histogram that was analyzed through the flow cytometer [23,24]. The hypo-diploid DNA content is an outcome from the activation of endogenous endonucleases in apoptotic cells that in turn causes DNA fragmentation [25].

Relative to the untreated control, treatment with incremental dosages of LF1 resulted in an increment of a distinct sub-G_1_ population in the DNA frequency histogram (Fig 2), which in turn can be interpreted as an increase in apoptotic effects towards the treated cells. This observation is consistent with the outcome from the MTT assay as well as AO/EB staining of LF1-treated cells. The sub-G_1_ population detection is a convenient assay in assessing the potential apoptotic capabilities of LF1, but has to be validated with tests that are specific towards the apoptotic markers. This leads us to the next phase of this study; the evaluation of apoptosis using the Annexin V /Propidium Iodide staining method.

Under normal physiological conditions, cells maintain an asymmetrical distribution of phospholipids such as phosphatidylserine (PS) between the inner and outer leaflet of the plasma membrane [26]. Apoptosis disrupts this asymmetric architecture which leads to the sustained presence of PS in the outer leaflet of the plasma membrane [27]. By utilizing the FITC-conjugated Annexin V, which has a specific affinity towards the PS region, this characteristic that is unique to the apoptotic process can be used to differentiate between viable and apoptotic cells. Moreover, the cell population can be further categorize into early apoptotic cells, secondary necrotic cells (late apoptotic cells) as well as necrotic cells through the concurrent usage of PI as an exclusion dye. This assay allows for a quantitative analysis into the occurrence of apoptosis in LF1-treated cells. Being an operator independent method, this method has the advantage of being more accurate as it is free from the user bias. Another advantage of this method is the increased statistical significance compared to more conventional methods such as microscopy assays, as it is possible to obtain a larger sample size (typically 10,000 or events).

The increasing presence of early and late apoptotic cells with the progression of time and increasing concentrations of treatment suggest that LF1 managed to induce a controlled demolition at the cellular level via the apoptotic pathway, as opposed to the un-programmed necrotic route, consistent with the data from the previous assays (MTT assay, AO/EB staining and Sub G_1_ population).

The cysteine-dependent aspartate driven proteases (caspases), which consist of several proteases, are major executors of apoptosis which are interconnected in a series to from a proteolytic cascade [28]. Caspases are typically divided into initiator caspases (caspase-2, -8, -9, and -10) which are activated upon interaction with adaptor protein, and the effector caspase (caspase-3, -6, and -7) that act directly on specific cellular substrate involved in the disablement of key proteins of the cell upon activation by the initiator caspases [29].

Among these enzymes, caspase-3 is a death protease that is frequently activated, triggering the specific cleavage of many key cellular proteins involved in apoptosis [28]. Activation of caspase-3 may occur through the intrinsic pathway which involves the release of cytochrome c and the initiator caspase-9 or via the extrinsic pathway that includes activation of death receptors as well as caspase-8 [29,30]. As such, caspase-3 is a perfect biomarker to affirm the role of LF1 in inducing apoptosis among treated cells as it is detectable in apoptotic cells regardless of the pathway taken.

The results obtain reflect an increasing presence of the activated caspase-3 enzyme, suggesting and a rise in the apoptotic activity in LF1 treated cells. Moreover, the dose-dependent characteristic exhibited by LF1 in the activated caspase-3 quantification is consistent with the data from the other apoptotic assessment within this study. Increment of LF1 dosage showed a similar trend of increased apoptotic activity in the Annexin V/PI assay and the sub-G_1_ quantification.

Caspase-3 plays a central role proteolytic cleavage of several proteins and is responsible for the apoptosis-associated chromatin condensation (observed in the AO/EB staining), DNA fragmentation (present in the Sub-G_1_ analysis), and nuclear collapse of the effected cells [31]. The elevated presence of active caspase-3 serves as a biochemical validation of the ability of LF1 in inducing apoptosis in treated lung cancer cells.

The growth development process in multicellular organisms is a complex process and need to be done in an appropriate and orderly fashion to avoid accumulated errors which may translate into unwanted situation, such as the formation of cancer cells. For this purpose, the cell employs a regulatory network of genes and proteins which serves as a check and balance in the development process to avoid formation of mutant cells. When this system fails or is bypassed due to external factors, the cell requires stimulants to activate the system in order to eliminate the defective (cancer) cell. Certain chemotherapeutic drugs disrupt the developmental process in the cancer cell by halting the cell cycle at a particular stage which results in the suppression of tumour proliferation before activating the apoptotic pathway leading to the demise of the cancer cells [32]. Our study thus far shows that LF1 is able to induce apoptosis in cancer cells. The cell cycle analysis was performed in the hopes of determining if cell death caused by LF1 through apoptosis involved arresting the cell cycle at a specific stage [33]. By doing so, the specific targets (genes or proteins) of LF1 can be narrowed and this in turns allows a better understanding into the nature of the semi-pure fraction.

The findings in this experiment suggest that LF1 managed to cause an arrest of the cell cycle at the G_0_/G_1_ phase in treated cells, thus preventing the transition from the G_1_ phase to the S phase leading to the demise of the cell. This argumentation also explains the decrease in the S and G_2_/M phase in both A549 and NCI-H1299 treated cells. As both the cell lines reacted similarly towards LF1, the role of the p53 gene can be ruled out by using the same line of reasoning mentioned in the MTT assay.

Possible targets of LF1 which may lead to the cell cycle arrest at the G_0_/G_1_ phase includes cyclin D and cyclin E as well as their corresponding cyclin-dependent kinase (CdK), CdK 2 and CdK4/6 [34]. Moreover, the p16 gene could also be a possible target of LF1 as the activation of this gene through cellular stress is known to inhibit the functionality of these kinases. Experimental data from the previous experimentation in this study has allowed us to rule out the p53 gene, which is usually associated with G_0_/G_1_ phase arrest, as a possible target of LF1. Based on these findings, the next step would be to evaluate if LF1 directly inhibits the activities of the kinases mentioned above or functionality of these kinases are prevented through the activation of the p16 gene.

## Conclusion

This study has shown that *L*. *flavescens* fraction, LF1 is capable of exerting cytotoxic effect on the tested lung carcinoma cells *via* the apoptotic pathway. The disruption of the cell cycle at the G_0_/G_1_ phase suggests LF1 may be targeting specific kinases or genes in the cells, channelling them into the apoptotic pathway. While it is still premature to suggest anti-tumour development from the constituents of *L*. *flavescens*, the potential of such an idea cannot be disregarded with the results obtained.

Nevertheless, a better understanding on the exact mechanism of how *L*. *flavescens* exerts its cytotoxic activity would be crucial, thus requiring further investigations.
